# *Gadus morhua* Eggs Sialoglycoprotein Prevent Estrogen Deficiency-Induced High Bone Turnover by Controlling OPG/RANKL/TRAF6 Pathway and Serum Metabolism

**DOI:** 10.3389/fnut.2022.871521

**Published:** 2022-04-12

**Authors:** Meihui Zhao, Fengfeng Mei, Jinfeng Lu, Qingying Xiang, Guanghua Xia, Xueying Zhang, Zhongyuan Liu, Chenghui Zhang, Xuanri Shen, Qiuping Zhong

**Affiliations:** ^1^Hainan Engineering Research Center of Aquatic Resources Efficient Utilization in South China Sea, School of Food Science and Engineering, Hainan University, Hainan, China; ^2^Collaborative Innovation Center of Provincial and Ministerial Co-construction for Marine Food Deep Processing, Dalian Polytechnic University, Dalian, China; ^3^Key Laboratory of Food Nutrition and Functional Food of Hainan Province, Hainan University, Haikou, China

**Keywords:** *Gadus morhua*, bone resorption, serum metabolism, osteoporosis, OPG

## Abstract

In recent years, the development of safe and effective anti-osteoporosis factors has attracted extensive attention. In this study, an estrogen-deficient osteoporosis rat model was employed to study the improving mechanism of sialoglycoprotein isolated from *Gadus morhua* eggs (Gds) against osteoporosis. The results showed that compared with OVX, Gds ameliorated the trabecular microstructure, especially the increased trabecular thickness, decreased trabecular separation, and enhanced the trabecular number. The analysis of qRT-PCR and western blotting found that Gds reduced bone resorption by inhibiting RANKL-induced osteoclastogenesis. The LC-MS/MS was used to investigate serum metabolism, and the enrichment metabolites were analyzed by the KEGG pathway. The results revealed that the Gds significantly altered the fat anabolism pathway, which includes ovarian steroidogenesis pathway and arachidonic acid metabolism pathway. Altogether, Gds could improve osteoporosis by suppressing high bone turnover *via* controlling OPG/RANKL/TRAF6 pathway, which is implicated with ovarian steroidogenesis pathway and arachidonic acid metabolism pathway. These findings indicated that Gds could be a candidate factor for anti-osteoporosis.

## Introduction

Osteoporosis is a metabolic disease induced by an imbalance in bone metabolism due to significant bone resorption than bone formation (1, 2). Estrogen can accelerate the differentiation of pre-osteoblasts, which stimulates bone collagen production and inhibits the activity of osteoclasts. Severe estrogen deficiency after menopause leads to the activation of osteoclast, increases bone conversion rate, affects calcium salt deposition, increases bone ablation and massive bone loss, decreases bone density, and finally leads to osteoporosis (3). At present, there are clinical drugs that can inhibit the activity of osteoclasts and reduce bone turnover to stabilize bone mineral density (BMD) and bone microstructure and improve osteoporosis, which includes estrogen-based drugs, bisphosphonates, denosulboxone, and renophene, etc. (4). However, long-term treatment with these drugs can cause a large number of side effects for patients. Thus, it is urgent necessity to identify the functional factors safely and effectively. Nutrition, which plays a crucial role in regulating osteoporosis (5) by decreasing bone loss *via* altering the body’s metabolism (6), is recommended as a complementary treatment to decrease the risk of osteoporosis (7).

As one of the most important economic fish, *Gadus morhua* produces about 30–50% byproducts during processing, which includes *Gadus morhua* eggs. *Gadus morhua* eggs contain all the nutrients needed for daily metabolic requirement development and are rich in unsaturated fatty acids, phospholipids, high phosphoprotein, glycoprotein, and other functional components. Our previous research found that *Gadus morhua* eggs contained sialoglycoprotein (Gds), which can increase bone formation and inhibit bone resorption. However, its’ *in vivo* mechanism has not been elucidated, which limits its further development and utilization.

RANKL/RANK/TRAF6 pathway is the key pathway in the regulation of osteoclast differentiation and activity (8, 9) and thereby adjusts bone resorption (10, 11). Factors such as parathyroid hormone and calcitriol can induce osteoblasts to produce receptor activator of nuclear factor (NF)-κB ligand (RANKL) and promote osteoclast formation through RANK receptor binding. After that, RANK interacts with TNF receptor-associated factors (TRAFs) that modulate the activation of phosphatidylinositol3-kinase (PI3K)-mediated NF-kappa B (NF-κB) pathways (12). At the same time, RANK can bind with several TRAFs to active RANK transmission to the downstream of NF-κB signal pathway, and the binding of RANKL to RANK recruits TRAF6 to stimulate the differentiation of osteoclast, which accelerates bone resorption and leads to osteoporosis.

Osteoprotegerin (OPG) is secreted out of the cell by osteoblast and antagonizes the osteoclast development induced by RANKL (10). Mesenchymal stem cells in bone marrow proliferate and differentiate to form osteoblasts (13). Osteoblasts secrete various osteogenic active substances, regulate extracellular matrix maturation, and promote bone formation. The signal transformation of bone marrow mesenchymal stromal cells differentiation in patients with osteoporosis *in vivo* results in increased adipocyte differentiation (14), decreased osteoblast differentiation, leading to lower expression of OPG, resulting in bone loss (15). The influence of adipocyte differentiation can be harnessed pharmacologically to osteoporosis treatment. Thus, understating the adipocyte synthesis metabolism *in vivo* is necessary.

Based on the close linkage among OPG/RANK/RANLK/TRAF6 pathway, osteoblast, and adipocyte, we treated the ovariectomized rats with Gds for 90 days, the liquid chromatography coupled with as spectrometry (LC-MS/MS) was used as an “omics” tool to reveal the specific metabolites associated with osteoporosis (16). We also observed the bone microstructure by micro-CT. After that, we further evaluated the bone resorption *via* determining the expression of OPG, RANKL, TRAF6 by western blotting and measuring the bone formation rate by MAR *via* calcium yellow-green labeling. Our main discovery displayed that the Gds could improve osteoporosis by altering the pathways involved in the fat synthesis KEGG pathway.

## Materials and Methods

### Materials

The *Gadus morhua* eggs were purchased from Guangzhou Meiyu Food Co., Ltd., (Guangzhou, China). TRAF6, OPG, RANKL, and β-actin antibodies were from Abcam (Cambridgeshire, United Kingdom). The primers of TRAF6, RANKL, OPG, and GADPH were synthesized by ShengGong Ltd., Co. (Shanghai, China). All chemicals and reagents used were of analytical grade.

### Preparation of Gds

The preparation of Gds was conducted according to Hei’s method (17). Gds contained 63.7% carbohydrate, 16.2% protein, and 18.6% *N*-acetylneuraminic acid with a molecular of 7,000-Da. The amino acid sequence was Ala-Ser-Asn-Gly-Thr-Gln-Ala-Pro with *N*-glycan on Asn, and *N*-glycan was composed of Man, GlcN, and Gal.

### Animal and Experimental Processor

There were 40 female Wistar (190 10 g) rats obtained from Changsha TianQin Biotechnology company (Changsha, China) with licensed ID: SCXK2014-011. The animal experiments had approved by the ethical committee of experimental animal care of Hainan University (HNDX2020072). All rats were housed at a constant temperature of 23°C with a 12-h light–dark cycling with food and water *ad libitum*.

After 1 week of acclimatization, rats were randomly divided into two groups (Sham-operated group, *n* = 8; ovariectomy group, *n* = 32). The animal experiment ovariectomized model and design methods refer to our previous research (3). After ovariectomy, successful osteoporotic model rats were defined as rats with serum E2 concentration significantly decreased and serum TRACP activity markedly increased in comparison with normal control group which underwent bilateral laparotomy. The ovariectomy group were randomly divided into 3 groups of 8 animals each (OVX, treated with physiological saline 1 ml/100 g.bw, E2, treated with 0.3 mg/kg.bw E2; Gds-L, treated with 200 mg/kg.bw Gds; Gds-H, treated with 400 mg/kg.bw). Meanwhile, the Sham group were intragastric 1 ml/100 g.bw physiological saline. The rats were received allocated administration once a day on the whole experiment, were anesthetized with 10% chloral hydras, and sacrificed by cervical dislocation (18).

To investigate mineral apposition rate (MAR), two rats were randomly selected from each group to be injected with calcein (5 mg/kg.bw) on days 13, 14, 3, and 4 before sacrifice. After 90 days of treatment, rats were euthanized. Serum was isolated by centrifuging with 3,000 *g* for 20 min at 4°C. The femurs and liver tissue were harvested and fixed in 10% methanol to measure biomechanical properties and bone histomorphology. The tibias were immediately isolated and stored at –80°C for qRT-PCR and western blotting analysis (19).

### Micro-CT Analysis

The distal metaphysis of the femur was imaged by a Micro-CT system. Parameters are as follows: at a voxel resolution of 20 μm; beam angel of increment: 0.5 a beam strength, 80 peak KV, 450 μA. The 3D images of coronal were constructed. The BMD, trabecular number (Tb.N), trabecular thickness (Tb.Th), and trabecular separation (Tb.Sp) were calculated by the tomography software (20).

### Histologic and Immunohistochemistry Evaluation

The decalcification-femurs were stained with H&E staining after soaking into 10% buffered formalin for 24 h, and the paraffin sections were 5.5 μm thick. The liver paraffine sections were handled with the 5.5 μm thick and stained with oil red staining. This part of the experiment was completed jointly by Wuhan Xavier Biotechnology Co., Ltd., (Wuhan, China) and the laboratory of Hainan University (Haikou, China). The pathological section was observed with a NIKON Eclipse CiDAP EX340-380 microscope (NIKON, Tokyo, Japan) at 50×, 400× magnification. In addition, bone sections labeled with calcite were directly observed under a microscope, and MAR was analyzed by ImageJ and Photoshop software.

### Detection of Urine Ca and P

Rats’ urine was collected continuously for 7 days in the morning before the last intragastric administration. After centrifugation (1,000 *g*, 10 min), the urine was stored at –80°C. Urine-related indexes Ca and P were detected by ELISA kit according to the instructions. Primary kits were purchased from Shanghai Enzyme Link Biotechnology Co., Ltd., (Shanghai, China).

### Quantitative Real-Time PCR Analysis

The bone tissue was taken out of the –80°C refrigerator and ground into fine powder in the automatic grinding machine (the temperature during grinding was controlled at 4°C). Then, the powder was put into a 2-ml EP tube and extracted total RNA from the bone in accordance with the instructions of the UNIQ-10 column animal total RNA extraction kit. The concentration and purity of total RNA (A260/A280 ratio) in bone tissue samples were determined by an ultra-micro nucleic acid analyzer (18, 21).

The first step is taking 2 μg RNA from each sample, adding 1.5 μl random primers, filling it with 12.5 μl sterile deionized water, and heating it at 65°C for 5 min. After cooling on ice, each tube was filled with the reverse transcriptase mixture from the 2 μl cDNA synthesis kit, 5 μl reverse transcriptase buffer, 0.5 μl RNA enzyme inhibitor, and 25 μl deionized water. The cDNA was obtained by cooling at 95°C for 5 min.

In this study, cDNA was amplified with a reaction system of 25 ml, 5 μl template cDNA, 12.5 μl SYBR Green fluorescent dye, 1.5 μl primer (0.75 μl upstream primer and +0.75 μl downstream primer), and 6 μl deionized water. Real-time PCR was performed at 95°C for 10 min, 95°C for 15 s, 60°C for 30 s, and 72°C for 30 s, and 40 cycles were amplified. GAPDH was used as the internal reference gene, and the relative expression level of target gene was expressed as the target gene expression level or internal reference gene expression level. Primer sequences of genes GAPDH, TRAF6, RANKL, and OPG are displayed in Supplementary Table 1.

### Western Blotting Analysis

The rat bone tissue was washed with phosphate buffer salt water for 3 times at 4°C and then ground into a fine powder with an automatic grinding machine. The powder was poured into a 2-ml EP tube, then 500 μl pyrolysis buffer was added, and let it stand on the ice for 20 min. The total bone solution was centrifuged at 10,000 *g* for 10 min, and the supernatant was collected. The protein concentration was determined with a BCA kit and adjusted to 30 ng/μl. The sample protein concentration was diluted with 5 × SDS-PAGE loading buffer and mixed gently, and then, the EP tube was placed in a water bath on the floating board and boiled for 10 min to denature the protein solution fully. The western blotting analysis was done according to the method of Mei et al. (3, 22).

### Serum Metabolism LC-MS/MS Analysis

The serum metabolism analysis was completed by the laboratory of Food College of Hainan University (Hainan, China) and Beijing Nohe Zhiyuan Co., Ltd., (Beijing, China). After obtaining the serum, we used LC-MS/MS to explore the composition of serum metabolites. The metabolomic analysis was performed on an ACQUITY UPLC CSH C18 column (100 mm × 2.1 mm, 1.7 μm) with acetonitrile: water (60:40) as mobile phase A and isopropanol: acetonitrile (90:10) as mobile phase B. The flow rate was 0.4 ml/min, and the elution procedure parameters are shown in Supplementary Table 2. MS parameter setting: capillary tube: 0.25 kV(+)/2 kV(-); sampling cone: 40 V; source temperature: 120°C; solvent removal temperature: 500°C; solvent removal/cone gas: 800/50 (L/ H); source offset: 80; ionization mode: ESI^+^/ESI^–^; TOF acquisition mode: resolution(+)/sensitivity(-); acquisition method continuous MSE; TOF quality range: 50–2,000 DA; scanning time: 0.1 s.

### Statistical Analysis

Metabolic data were analyzed by CD search library software, (raw), retention time and mass ratio of parameters such as charge simple filtering, and then, according to the retention time of different samples, deviation 0.2 min and quality deviation of peak aligned 5 PPM, make the evaluation more accurate, then according to set up the quality deviation of 5 PPM, signal strength is 30%, the signal-to-noise ratio of 3, 1,00,000 minimum signal strength, and ion peak, such as information extraction, and peak area for quantitative, to integrate target ion. Then, molecular formula prediction is carried out through molecular ion peaks and fragment ions and compared with mzCloud^1^, mzVault, and Masslist database. Background ions are removed with blank samples, quantitative results are normalized, and finally, data identification and quantitative results are obtained.

Other results’ values were expressed as mean ± SD Statistically significant differences were evaluated by ANOVA and *t*-test using SPSS software. Values of *p* < 0.05 were considered statistically significant.

## Results

### Gds Protected Rats Against the Bone Loss Caused by Estrogen Deficiency

**FIGURE 1:**
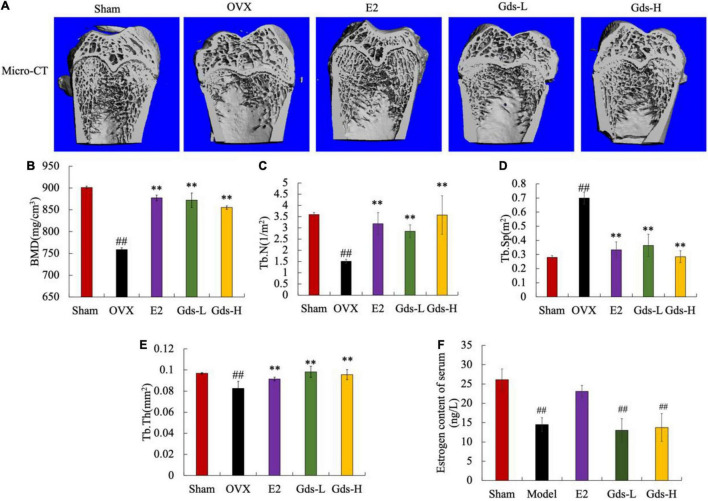
Gds protected rats against the bone loss caused by estrogen deficiency. **(A)** Representative micro-CT image of trabecular region of each group; **(B)** BMD (mg/cm^3^), bone mineral density; **(C)** Tb.N (1/m^2^), trabecular thickness; **(D)** Tb.Sp (m^2^), trabecular separation; **(E)** Tb.Th (mg/cm^2^), trabecular thickness; **(F)** estrogen (ng/L). Sham, sham operated group; OVX, ovariectomized control group; Gds-L, low dose of *Gadus morhua* sialoglycoprotein-treated group; Gds-H, high dose of *Gadus morhua* sialoglycoprotein-treated group; E2, positive drug control group. Ave ± SE, ^#^*p* < 0.05, ^##^*p* < 0.01 vs. Sham group; *p < 0.05, ^**^p < 0.01 vs. OVX group. *n* = 8.

We analyzed the bone mass of the distal femur of rats by micro-CT and evaluated the effect of Gds treatment on the improvement of bone microstructure in rats. As shown in Figure 1A, the femoral microstructure of OVX was seriously damaged, the trabecular region was thin and sparse, and the three-dimensional structure was seriously damaged. However, Gds and E2 significantly improved the microstructure of bone trabeculae, and the network structure of bone trabeculae was well repaired.

Bone mineral density is closely related to osteoporosis and is a key criterion for the diagnosis of osteoporosis. As shown in Figure 1B, the BMD of the Sham group was 859.378 ± 30.464 mg/cm^3^, and that of the OVX group was 444.28 ± 3.875 mg/cm^3^. Compared with Sham group, the BMD in OVX group was significantly decreased (*p* < 0.01). E2, Gds-L, and Gds-H significantly increased the BMD of rats to 877.105 ± 6.503 mg/cm^3^, 869.008 ± 31.464 mg/cm^3^, and 851.071 ± 3.179 mg/cm^3^, respectively. These results indicated that Gds and E2 could significantly improve BMD in osteoporosis rats.

Further results of quantitative data on bone trabecular structure showed that compared with Sham group, the Tb.N in the OVX rats was significantly reduced (Figures 1A,C), the Tb.Sp was significantly increased (Figures 1A,D), and the Tb.Th was significantly attenuated (Figures 1A,E), which indicates that the dense network structure of bone trabeculae was destroyed. Gds and E2 significantly increased the Tb.N and Tb.Th, decreased the Tb.Sp, and improved the damaged bone microstructure. In addition, as shown in Figure 1F, estrogen levels in OVX and Gds groups were significantly lower than those in Sham group after ovariectomy except the E2 treatment group, which indicates that Gds can improve the damage of bone microstructure in estrogen deficiency rats, but do not have estrogen-like effect.

### Gds Decreased High Bone Turnover Led by Estrogen Deficiency

**FIGURE 2:**
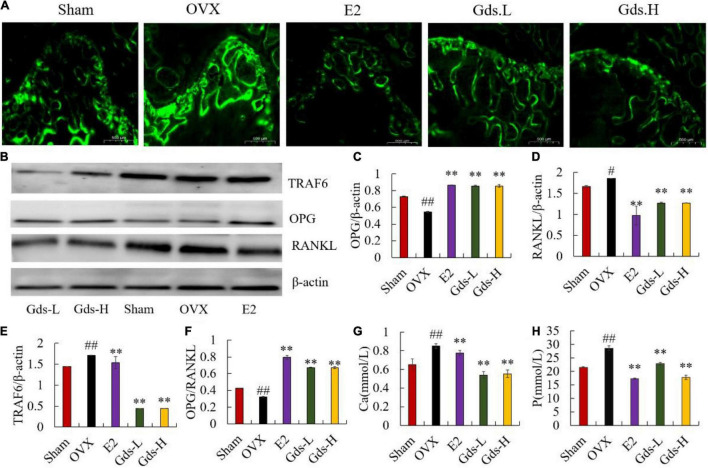
Gds protected rats against the high bone turnover leaded by estrogen deficiency. **(A)** Representative fluorescence labeling results of calcein in femoral of each group; **(B)** the western blot image showed the expression of **(E)** TRAF6, **(C)** OPG, **(D)** RANKL; expression of bone and the quantified content of protein were analyzed by ImageJ. **(F)** OPG/RANKL, **(G)** Ca, the amount of Ca ions in urine (mmol/L), **(H)** P, the amount of P ions in urine. Sham, sham operated group; OVX, ovariectomized control group; Gds-L, low dose of *Gadus morhua* sialoglycoprotein-treated group; Gds-H, high dose of *Gadus morhua* sialoglycoprotein-treated group; E2, positive drug control group. Ave ± SE, ^#^*p* < 0.05,^##^*p* < 0.01 vs. Sham group; *p < 0.05, ^**^p < 0.01 vs. OVX group.

The previous studies have shown that postmenopausal osteoporosis is due to estrogen deficiency resulting in bone resorption significantly exceeded than bone formation and is a high bone turnover model (23, 24). Calcein binds preferentially to calcium by chelation and can be used to investigate the inhibitory effect of Gds on hyper bone transformation. We evaluated new bone formation and MAR in the femur of rats by calcein labeling. In histological slides (Figure 2), the distance between bright green fluorescent double lines was used to show the new bone formation. The amount of new bone formation in OVX was significantly increased than that in Sham, while the distance between the two lines decreased after Gds treatment (Figure 2A). MAR of Sham (5.660 ± 0.818 μm/d) and E2 (5.904 ± 0.0734 μm/d) was significantly lower than that of OVX (21.042 ± 1.20 μm/d, *p* < 0.01) (Supplementary Figure 1). Compared to the OVX group, Gds inhibited high bone formation, in both low and high doses (MAR of Gds-L, 12.38463 ± 1.209 μm/d, *p* < 0.01; MAR of Gds-H, 10.116 ± 1.306 μm/d, *p* < 0.01) (Supplementary Figure 1), which reflects a positive effect against high bone turnover (19).

Increasing bone resorption is responsible for estrogen deficiency-induced osteoporosis. OPG/RANKL system plays an important role in the bone resorption (10, 25, 26). As a member of the TNF family, RANKL can bind to RANK in the surface of osteoclast, recruit the adaptor TRAF6 to activate the osteoclast, and increase bone resorption and bone loss (27–30). However, OPG can competitively inhibit the binding of RANKL and RANK and suppress bone resorption. As the osteoporosis model constructed by bilateral ovariectomy was a high bone turnover model, OPG significantly increased in OVX at the gene level (Supplementary Figure 2B), and the content of OPG in the E2 and Gds group was significantly higher than that in the OVX group. Interestingly, the western blotting results showed that the OPG was significantly decreased in OVX and enhanced after Gds and E2 treatment (*p* < 0.001) (Figures 2B,C). Both the gene and the protein expression level displayed that the RANKL in femur of ovariectomized rats was significantly increased (*p* < 0.001), while Gds prevented the increase in RANKL level in ovariectomized rats (*p* < 0.001) (Figures 2B,D and Supplementary Figure 2C). Further analysis showed that OPG/RANKL values were significantly decreased in OVX, and Gds and E2 intake significantly increased OPG/RANKL values in the femur (*p* < 0.001) (Figures 2B,F). Estrogen deficiency significantly increased the expression of TRAF6 in the OVX group, whereas Gds significantly decreased the expression of TRAF6 (Supplementary Figure 2A and Figures 2B,E), which indicates that Gds can inhibit the expression of TRAF6 and suppress the activation of osteoclast.

The content of Ca and P ions in urine is an intuitive indicator of bone loss. Compared with the Sham group, the contents of urinary Ca and P were significantly increased in the OVX group, while the contents of urinary Ca and P were significantly decreased by Gds group (*p* < 0.01), which indicates that Gds can reduce the activity of osteoclast and inhibit bone resorption. All in all, these results suggest that Gds can improve osteoporosis by suppressing the activation of osteoclast and inhibiting bone resorption.

### Gds Reduced Bone Loss by Regulating Lipid Metabolism

**FIGURE 3:**
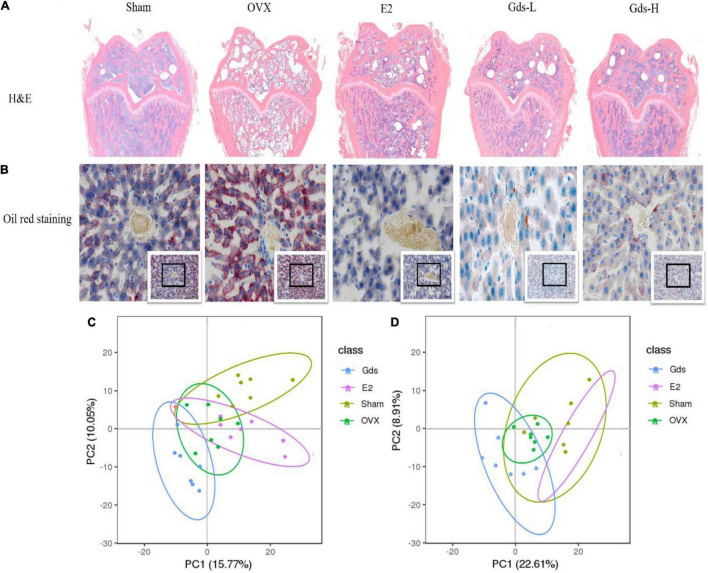
Gds protected rats against fat anabolism leaded bone loss by regulating lipid metabolism. **(A)** Histological image of bone was displayed by H&E staining. **(B)** The lipid level of liver was showed by oil red staining. **(C)** The serum metabolism score plot of PCA for each group in positive ion mode. **(D)** The serum metabolism score plot of PCA for each group in positive ion mode. Sham, sham operated group; OVX, ovariectomized control group; Gds-L, low dose of *Gadus morhua* sialoglycoprotein-treated group; Gds-H, high dose of *Gadus morhua* sialoglycoprotein-treated group; E2, positive drug control group.

The previous studies found that the increased bone resorption, extensive bone resorption, would lead to bone loss, bone microstructure destruction, affect the differentiation of mesenchymal cells into osteoblasts, inhibit the maturation of osteoblasts, and promote the differentiation of mesenchymal cells into adipocytes to form bone fat (31, 32). H&E staining was used to analyze the changes in femur tissue structure and evaluate the effect of Gds on the improvement of bone texture morphology. As shown in Figure 3A, compared with Sham group, the femoral structure of OVX was severely damaged with significant bone loss, bone density was significantly decreased, and intertrabecular fat was significantly accumulated. However, Gds and E2 significantly improved femoral structure, reduced bone loss, decreased interosseous fat, and repaired damaged bone tissue structure.

Bone loss results from the increasing adipocyte differentiation and decreasing osteoblast differentiation in bone marrow in patients. This study showed that the femur of the OVX group contained more fat and lower BMD, and Gds and E2 had inhibitory effects on fat formation, which indicates that the fat metabolism in the OVX group was significantly changed compared with Sham group. The liver is one of the main organs of lipid metabolism (33, 34). The morphology of the liver was further observed by oil production red staining in this experiment. As shown in Figure 3B, after ovariectomy, the hepatic portal vein in the OVX group was enlarged and the blue-stained nucleus was reduced, which indicates the decrease of normal liver cells and increased necrosis of liver cells. The number of adipocytes stained with oil red was significantly increased in OVX group, which was significantly decreased after treatment with Gds, which indicates that Gds changed the anabolism of liver fat in ovariectomized rats. From these pathological analyses, we speculated that Gds could prevent liver lipid metabolic disorders in ovariectomized rats.

Serum metabolism analysis is an analysis of multivariate data sets, which reflects the total metabolic difference between each group and the variation degree between the samples within the group. LC-MS/MS was used to detect the total metabolic diversity of serum in Sham, OVX, E2, and Gds-L group (Gds-L was confirmed as the optimal dose through the analysis of bone tissue structure, liver morphology, and fat distribution) to verify the relationship among Gds, fat metabolism, and osteoporosis. Principal component analysis (PCA) profiles showed that the serum metabolites of Gds, E2, Sham, and OVX were segregated clearly (Figures 3C,D). In the positive ion model with PCA, there were 660 general identified metabolites. Compared with Sham, the serum metabolites of OVX were significantly changed, which includes the 47 downregulated metabolites (*p* < 0.01) and 41 upregulated (*p* < 0.01) metabolites. The significant difference in metabolites between Gds and OVX was 102 (*p* < 0.01), which includes the 36 upregulated and 66 downregulated metabolites. In the negative ion model with PCA, there were 311 general identified metabolites (Table 1). Compared with Sham, the serum metabolites of OVX were significantly changed, which includes the 8 downregulated metabolites (*p* < 0.01) and 28 upregulated metabolites (*p* < 0.01). The significant difference in metabolites between Gds and OVX was 69 (*p* < 0.01), which includes the 28 upregulated and 41 downregulated metabolites. Collectively, these results shown that the Gds leads an altered serum metabolite associated with osteoporosis in ovariectomized rats.

**TABLE 1:** Result of metabolite difference screening.

	Compared Samples	Num. of Total Ident.	Num. of Total Sig.	Num. of Sig. Up	Num. of Sig. down
Positive model	OVX vs. Sham	660	88	41	47
	E2 vs. OVX	660	98	20	78
	Gds vs. OVX	660	102	36	66
	ACP vs. E2	660	163	95	68
Negative model	OVX vs. Sham	311	36	28	8
	E2 vs. OVX	311	69	11	58
	Gds vs. OVX	311	69	28	41
	Gds vs. E2	311	103	71	32

*Differential metabolites were screened. p-value is calculated by t-test, which indicates the level of difference significance. The threshold was set as p-value < 0.05.*

### Gds Decreased Fat Synthetic *via* the Metabolic Signal Pathway

**FIGURE 4:**
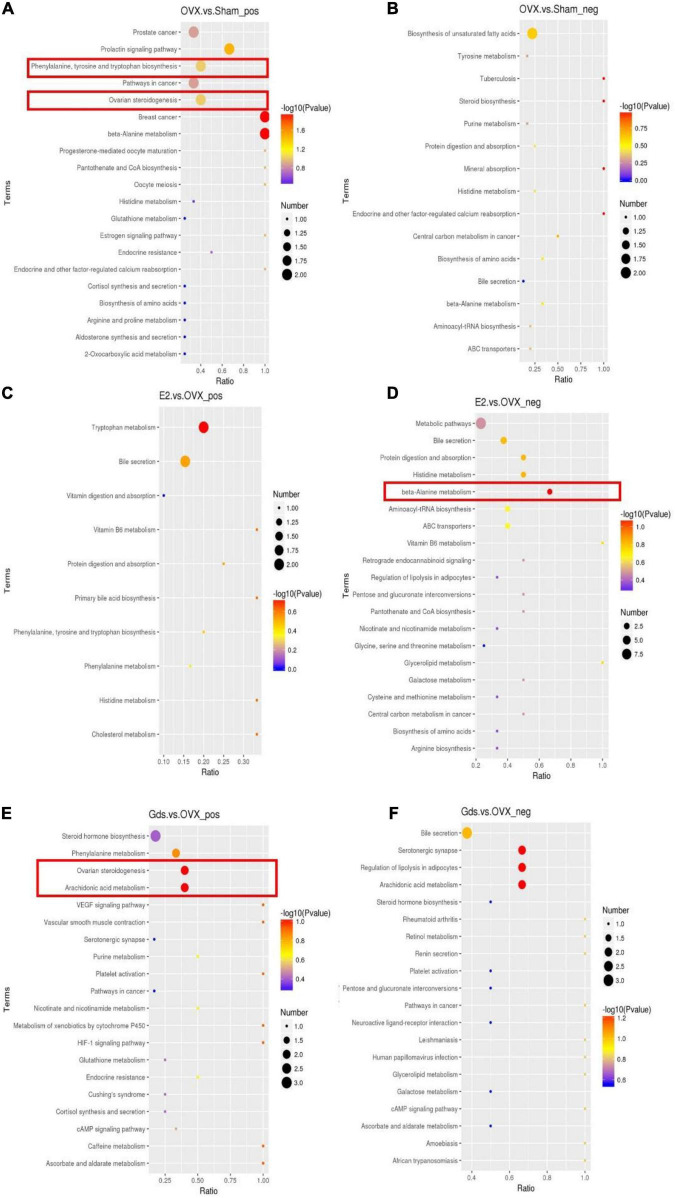
Gds protected ovariectomized rats against the increasing of fat synthetic *via* metabolic signal pathway. **(A)** Top 20 results of KEGG enrichment signal pathway at positive ion model of OVX. vs. Sham; **(B)** top 20 results of KEGG enrichment signal pathway at negative ion model of OVX. vs. Sham; **(C)** top 20 results of KEGG enrichment signal pathway at positive ion model of E2. vs. OVX; **(D)** top 20 results of KEGG enrichment signal pathway at negative ion model of E2. vs. OVX; **(E)** top 20 results of KEGG enrichment signal pathway at positive ion model of Gds. vs. OVX; **(F)** top 20 results of KEGG enrichment signal pathway at negative ion model of Gds. vs. OVX. Sham, sham-operated group; OVX, ovariectomized control group; Gds, *Gadus morhua* sialoglycoprotein-treated group; E2, positive drug control group.

In recent years, several reports have demonstrated that the detail pathway associated with the function of bone resorption of metabolism can be reflected by metabolites. To address the causation for the increase of fat synthesis after ovariectomy and investigate the relationship among estrogen deficiency, lipid synthesis, and osteoporosis, we analyzed the functional types of significant enrichments by the Kyoto Encyclopedia of Genes and Genomes (KEGG) pathways. The bubble diagram (Figure 4) displayed the functional classification and pathways of the top 20 enriched proteins of different compared groups in positive and negative ion models, respectively. The significant classifications are framed in red (*p* < 0.05).

The enrichment result of OVX vs. Sham at positive ion model demonstrated that phenylalanine, tyrosine, and tryptophan biosynthesis pathways and ovarian steroidogenesis pathway were enriched in the OVX group (Figure 4A) and no statistically significant change at negative ion model (Figure 4B). In phenylalanine, tyrosine, and tryptophan biosynthesis pathways, compared with Sham, the serum content of indole and phenylalanine was upregulated and downregulated in the OVX group, respectively (Supplementary Figure 3). The previous studies showed that indole was involved in tryptophan metabolism associated with bone mass *via* 5-HT (35, 36). 5-HT can inhibit the formation of osteoblasts and increase the activities of osteoclasts, thereby promoting bone loss. The metabolism and synthesis of L-tryptophan and D-tryptophan in OVX are very fast, and a large amount of indole is used to synthesize 5-HT (37), which finally increased bone loss. Serum phenylalanine levels are associated with oxidative stress status *in vivo*, nitric oxide metabolism, cholesterol-derived oxidative sterols, vitamin D, and bone status (38). The low phenylalanine level in OVX may also be one of the causes of osteoporosis. In ovarian steroidogenesis pathway, the progesterone and 17-β-estradiol were decreased in the OVX group (Supplementary Figure 4). Both progesterone and 17-β-estradiol are associated with estrogen metabolism, and in the absence of specific drug intake, progesterone and 17-β-estradiol in the OVX group were normally reduced.

The enrichment analysis of E2 vs. OVX showed that a significant majority of beta-alanine metabolism at the negative model (Figure 4D) that leads histidine and L-aspartate decreased in E2 group (Supplementary Figure 5). However, there was no statistically significant change in the positive ion model (Figure 4C). The histidine and L-aspartate, which decreased after E2 group, are associated with osteoarthritis (39) and liver function (40), which indicates that the E2 protected bone mass *via* histidine and L-aspartate metabolism.

The following 2 significantly different KEGG pathways were enriched between Gds vs. OVX, which includes ovarian steroidogenesis and arachidonic acid metabolism pathway in positive ion model (Figure 4E). In ovarian steroidogenesis pathway, compared with OVX, Gds decreased the level of 17-hydroxy-pregnenolone and testosterone (Supplementary Figure 6). The 17-hydroxy-pregnenolone, testosterone, PGB_2_, and prostacyclin were related to the sterol and arachidonic acid metabolism (41, 42). In arachidonic acid metabolism pathway, compared with OVX group, Gds downregulated the expression of PGB_2_ and prostacyclin (Supplementary Figure 7). From those KEGG analysis, we hypothesize that the improvement effect of Gds against osteoporosis was implicated in the altered serum metabolism.

## Conclusion

This study revealed that Gds could reduce bone resorption, increase bone formation, suppress high bone turnover, and improve osteoporosis in ovariectomized rats *via* OPG/RANKL signal axis, which is closely related to the serum metabolism. Thus, Gds could be a candidate factor of anti-osteoporosis.

## Data Availability Statement

The original contributions presented in the study are included in the article/Supplementary Material, further inquiries can be directed to the corresponding author/s.

## Ethics Statement

The animal study was reviewed and approved by HNDX2020072.

## Author Contributions

MZ and FM: project administration, methodology, software, validation, formal analysis, data curation, and writing—original draft preparation. JL, QX, XZ, and ZL: writing, reviewing, and editing. CZ and XS: conceptualization and investigation. GX and QZ: visualization, supervision, funding acquisition, and resources. All authors have read and agreed to the published version of the manuscript.

## Conflict of Interest

The authors declare that the research was conducted in the absence of any commercial or financial relationships that could be construed as a potential conflict of interest.

## Publisher’s Note

All claims expressed in this article are solely those of the authors and do not necessarily represent those of their affiliated organizations, or those of the publisher, the editors and the reviewers. Any product that may be evaluated in this article, or claim that may be made by its manufacturer, is not guaranteed or endorsed by the publisher.

## Footnotes

1
https://www.mzcloud.org/
