# Supplementary material for: Gadus morhua Eggs Sialoglycoprotein Prevent Estrogen Deficiency-Induced High Bone Turnover by Controlling OPG/RANKL/TRAF6 Pathway and Serum Metabolism
Source: Front Nutr. 2022 Apr 12;9:871521. doi: 10.3389/fnut.2022.871521 (PMC9040668; doi:10.3389/fnut.2022.871521)
Supplement: Supplementary file 1 [file Data_Sheet_1.docx]

Supplementary table 1. Primer used for the real-time polymerase chain reaction (Q-PCR).

| Gene | Sequence (5’-3’) |
| --- | --- |
| TRAF6 | F- GCGCTGTGCAAACTACATTT |
|  | R- AAGGATCGTGAGGCGTATTG |
| RANKL | F- AGGCTGGGCCAAGATCTCTA |
|  | R- GATAGTCCGCAGGTACGCTC |
| OPG | F- GGCAGGGCATACTTCCTGTT |
|  | R- GCCACTTGTTCATTGTGGTCC |
| GADPH | F-TGCCACTCAGAAGACTGTGG |
|  | R-TTCAGCTCTGGGATGACCTT |

Supplementary table 2 Elution parameters

| Time | A | B |
| --- | --- | --- |
| 0～2 min | 60% | 40% |
| 2~2.1 min | 57% | 43% |
| 2.1~6 min | 50% | 50% |
| 6～6.1 min | 46% | 54% |
| 6.1～8 min | 30% | 70% |
| 8~8.1 min | 1% | 99% |
| 8.1~10 min | 60% | 40% |

Figure S1


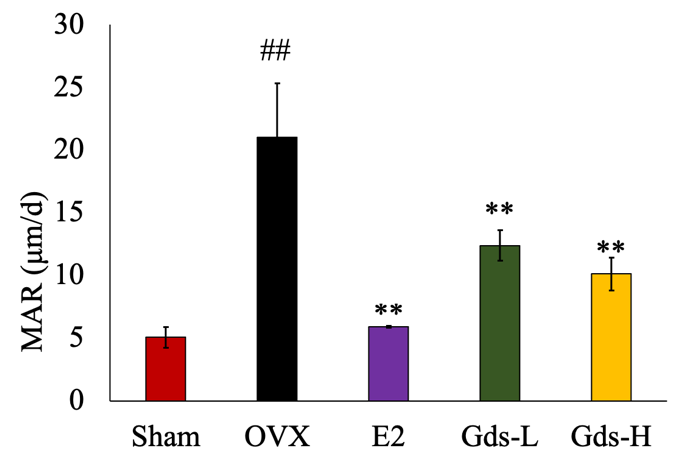


Figure S1 MAR in the femur of rats. Sham, sham operated group; OVX, ovariectomized control group; Gds-L, low dose of *Gadus morhua* Sialoglycoprotein treated group; Gds-H, high dose of *Gadus morhua* Sialoglycoprotein treated group; E2, positive drug control group. Ave ± SE, ^#^*P*< 0.05，^##^*P*< 0.01 versus Sham group; ^*^*P*< 0.05, ^**^*P*< 0.01 versus OVX group.

Figure S2


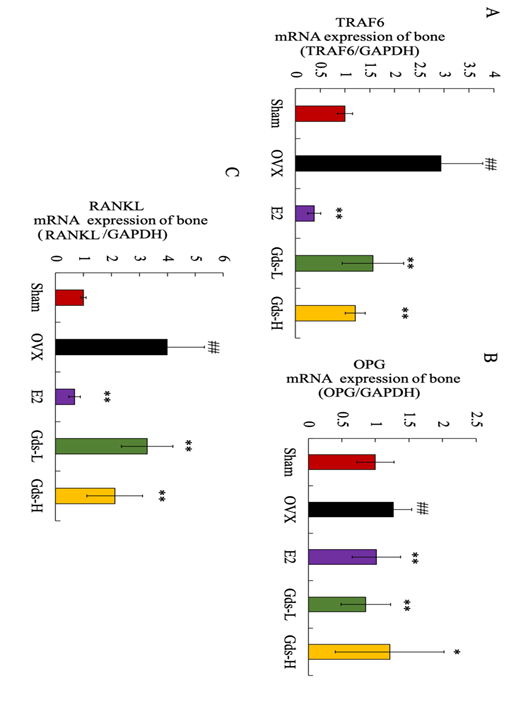


Figure S2 The mRNA expression of (A) TRAF6, (B) OPG, (C)RANKL in bone. Sham, sham operated group; OVX, ovariectomized control group; Gds-L, low dose of *Gadus morhua* Sialoglycoprotein treated group; Gds-H, high dose of *Gadus morhua* Sialoglycoprotein treated group; E2, positive drug control group. Ave ± SE, ^#^*P*< 0.05，^##^*P*< 0.01 versus Sham group; ^*^*P*< 0.05, ^**^*P*< 0.01 versus OVX group.

Figure S3


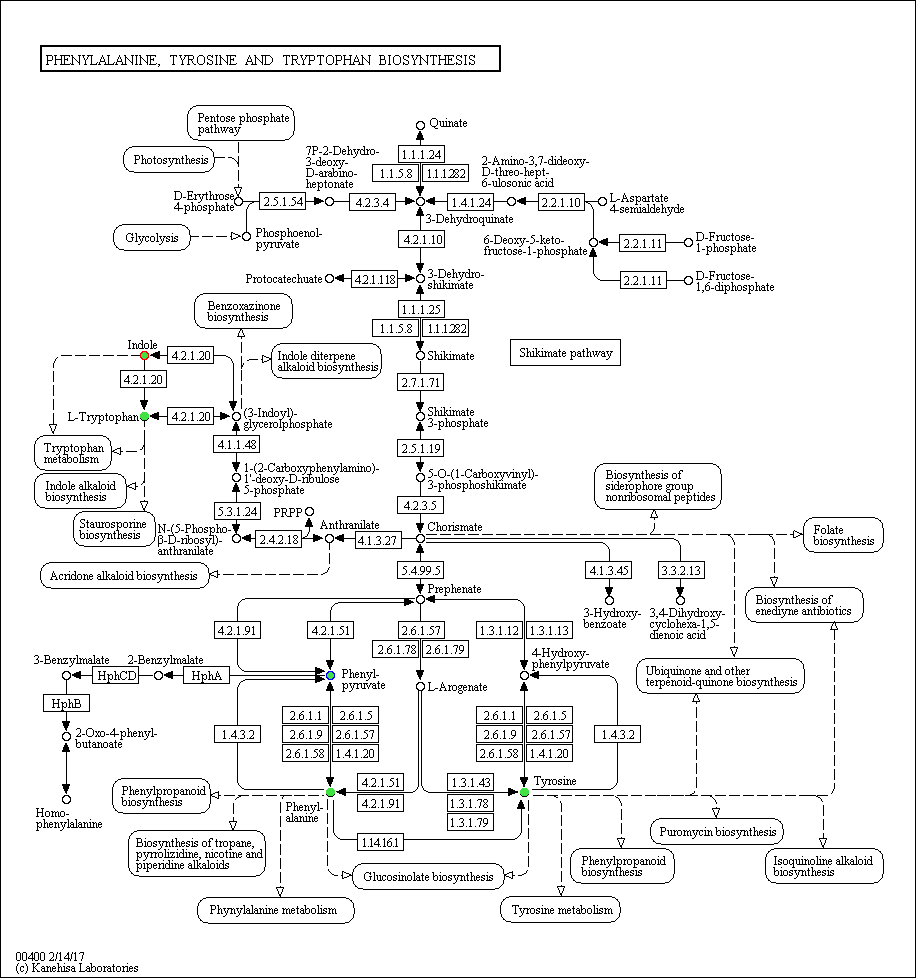


Figure S3 Phenylalanine, tyrosine and tryptophan biosynthesis pathway

Figure S4


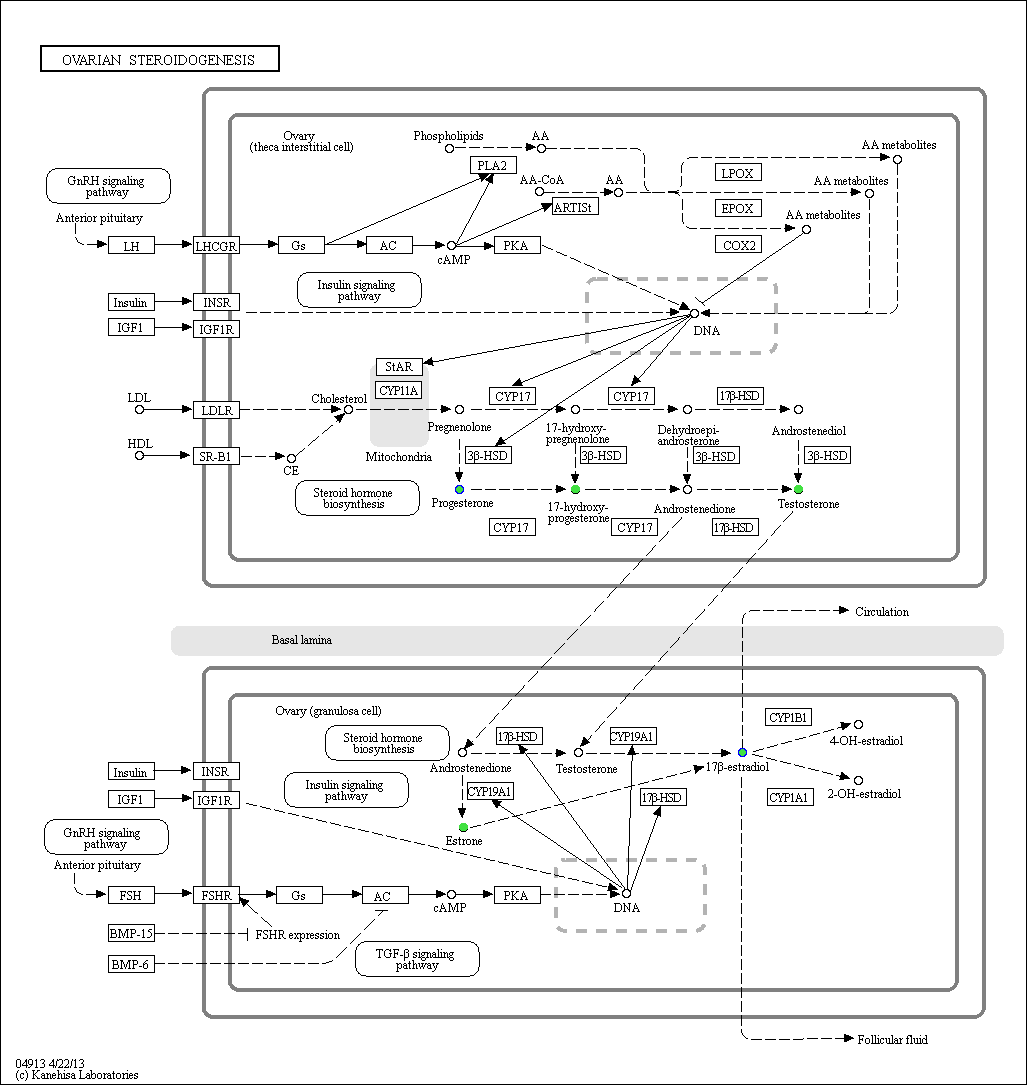


Figure S4 Ovarian steroidogenesis pathway

Figure S5


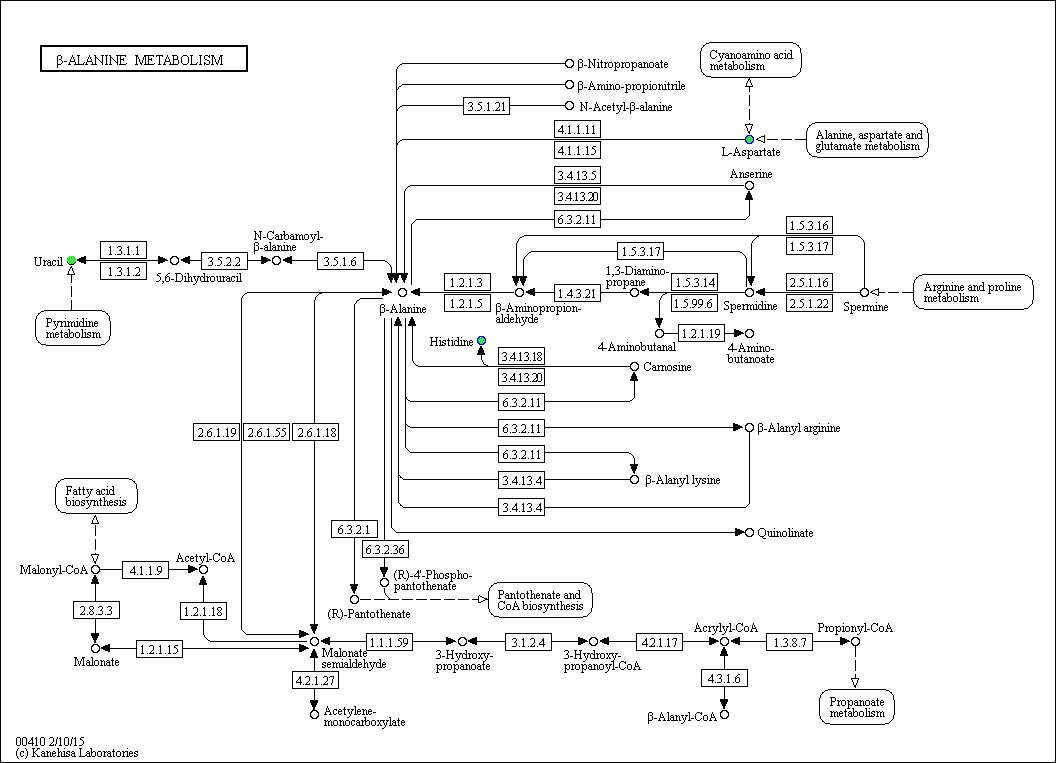


Figure S5 β-alanine metabolism

Figure S6


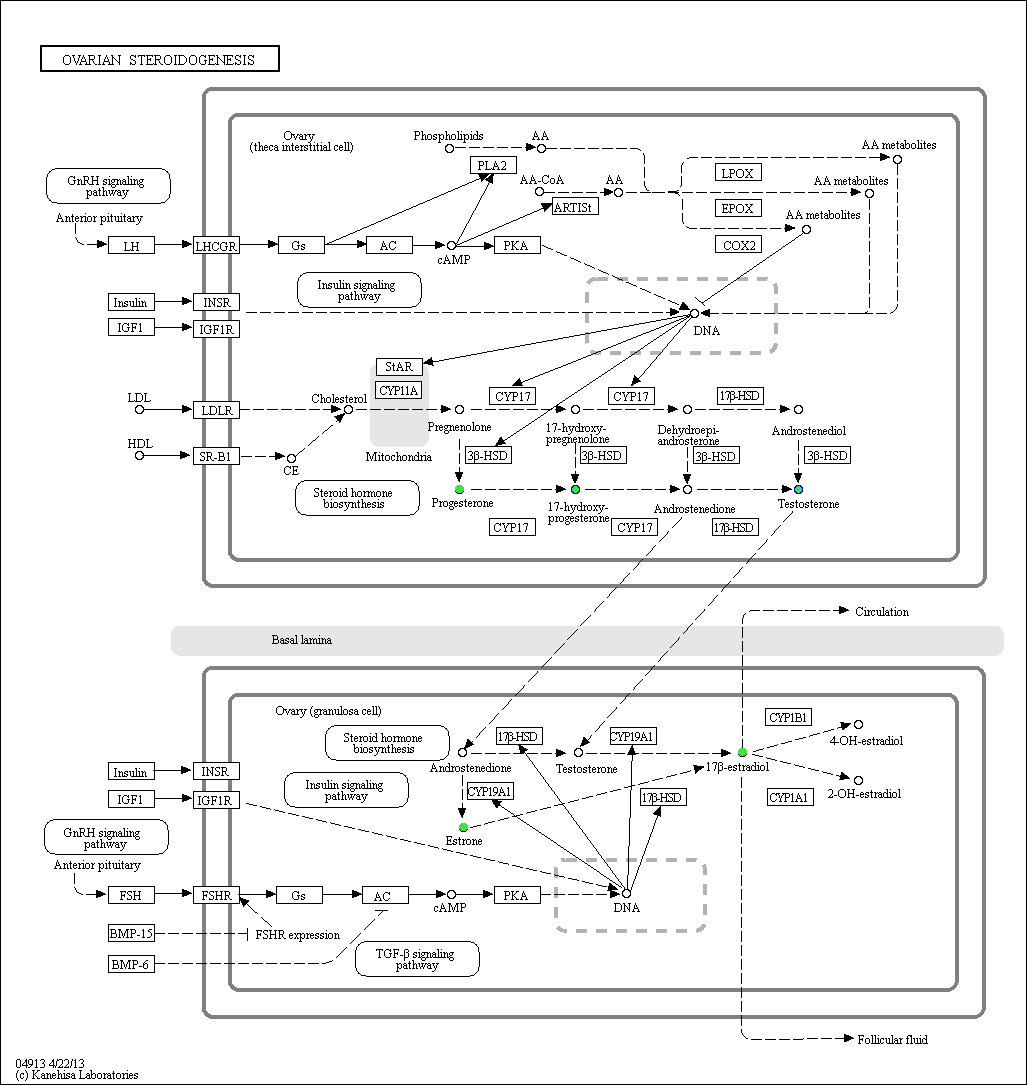


Figure S6 Ovarian steroidogenesis

Figure S7


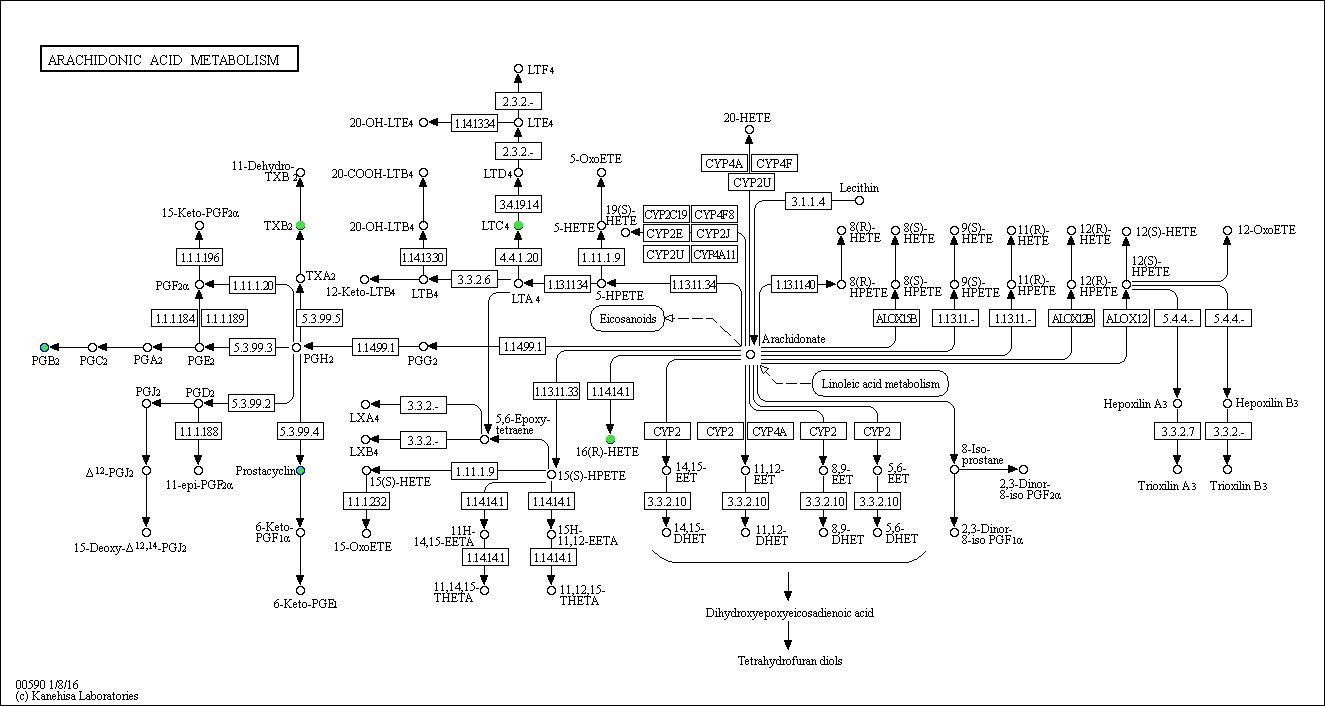


Figure S7 Arachidonic acid metabolism
